# Observer-Based Source Localization in Tree Infection Networks via Laplace Transforms

**DOI:** 10.1007/s11538-026-01640-4

**Published:** 2026-04-27

**Authors:** Graham Kesler O’Connor, Julia M. Jess, Devlin Costello, Manuel E. Lladser

**Affiliations:** Department of Applied Mathematics, University of Colorado, Boulder, CO 80309-0526 US

**Keywords:** Diffusion source, Graph, Infection propagation, Information diffusion, Laplace estimation, Rumor spreading, SI model

## Abstract

We address the problem of localizing the source of infection in an undirected, tree-structured network under a susceptible–infected outbreak model. The infection propagates with independent random time increments (i.e., edge-delays) between neighboring nodes, while only the infection times of a subset of nodes can be observed. We show that a reduced set of observers may be sufficient, in the statistical sense, to localize the source and characterize its identifiability via the joint Laplace transform of the observers’ infection times. Using the explicit form of these transforms in terms of the edge-delay probability distributions, we propose scale-invariant estimators of the source. We evaluate their performance on synthetic trees and on a river network, demonstrating accurate localization under diverse edge-delay models.

## Introduction

Interest in analyzing and understanding large-scale infections has persisted for decades. Extensive research has explored how infections grow and evolve as they spread across networks (William et al. 1927; Roy et al. 1992; Leinhardt 2013; Wasserman and Faust 1994; Mollison 1977; Newman 2002; Eubank et al. 2004). In contrast, source localization has received significantly less attention, despite the fact that identifying an infection source quickly and accurately is crucial for containment and the prevention of issues such as disease outbreaks and the spread of misinformation or contaminants.

In recent years, various observer-based solutions have been proposed for the source localization problem. These methods, developed in Pinto et al. (2012); Paluch et al. (2021); Shen et al. (2016), use the infection times of a typically sparse subset of nodes in an infection network to try to identify the source. To the best of our knowledge, Pinto, Thiran, and Vetterli introduced the first observer-based approach in 2012 Pinto et al. (2012). They employ a maximum likelihood estimator (MLE) derived from the joint probability density function (p.d.f.) of the observers’ infection times and show that the MLE is optimal when the infection propagates over a tree—i.e., an undirected, connected graph without cycles—with independent but not necessarily identically distributed Gaussian propagation delays along the edges. Concrete examples of infections propagating over a tree-like network include waterborne pathogens propagating downstream through river networks (for example, due to a cholera-contaminated fecal deposit); biohazardous materials spreading through a building’s water or ventilation pipelines; and rumors cascading through a compartmentalized hierarchical organization.

Due to the complex interdependencies among the paths connecting the source and the observers in a tree, the MLE of the source under Gaussian delays remains the only case where an analytic expression for the joint distribution of observers’ infection times is known. Nevertheless, the time complexity of this approach is linear in the number of nodes in the tree and it can be reduced by excluding observers with relatively large infection times (Paluch et al. 2021). An alternative approach to source localization is based on least squares, minimizing over all non-observer nodes the sum of squared differences between the observed and expected infection times of the observers (Shen et al. 2016).

In practice, most networks through which an infection propagates—whether representing physical social interactions, contacts in online platforms, or computer networks—contain cycles, allowing transmission along a usually exponentially large number of possible paths between a source and each node. Nonetheless, the tree structure is technically appealing, particularly in susceptible-infected (SI) models, where the infection propagates along a growing tree that eventually spans the whole network. Because of this, source localization methods typically assume that the infection propagates along a spanning tree of the network. The criteria for selecting this tree vary widely in the literature, ranging from simple breadth-first search trees (Pinto et al. 2012), to shortest path trees (Paluch et al. 2021), to convex linear combinations of Gromov matrices (Ji et al. 2019), among others.

**Paper organization.** In the remainder of the Introduction, we introduce details and notation for the tree infection model addressed in this work. In Section 2, we show how to identify redundant observers, reducing the source estimation problem to tree networks in which the observers are leaves, except possibly for a single observer. In Section 3, we address the identifiability of the source in terms of the Laplace transform of the vector of observers’ infection times. We then use the explicit form of this transform in Section 4 to propose two source estimators based on the empirical Laplace transform of the observers’ infection times. Section 5 is devoted to test our methods both in synthetic networks and an existing river network under various practical edge-delay models. Finally, Section 6 presents concluding remarks, and Section 7 contains the technical proofs of some of our preceding results.

This work is partially based on results and ideas from the recent theses (O’Connor 2022; Jess 2024).

The implementation of all methods discussed in this manuscript, and the synthetic and real networks used to support our findings can be found in the GitHub repository: Costello (2025).

### Infection Model

We assume an infection propagates between neighboring nodes in a fixed tree with *vertex set* *V* and *edge set* *E*. Edges are undirected. The tree $T=(V,E)$ is known, finite, and undirected. A *leaf* in *T* is a node with precisely one neighbor (i.e., a node of degree 1). We denote the *leaf set* of *T* as *L*.

For nodes $u,v \in V$, we use [*u*, *v*] to represent, depending on the context, the set of edges or vertices on path connecting *u* and *v*. This path is unique because *T* is a tree.

The infection is assumed to begin at time zero from a single unknown node. We model its spread using an SI model originating at a node $s\in V$—the unknown source. Since SI models do not allow recovery, the infection continues to spread until every node in *T* becomes infected. We assume that for each edge $e=\{u,v\}\in E$, the infection propagates from an already infected node *u* to a susceptible neighbor *v* after a non-negative, random amount of time (or delay) having a known continuous probability distribution. We denote this delay as $\tau _e$. The random variables $\tau _e$, with $e\in E$, are assumed independent.

For each $v\in V$, define

$$\begin{aligned} \tau _v := \sum _{e\in [s,v]} \tau _e. \end{aligned}$$

In other words, $\tau _v$ is the time of infection of node *v*. More generally, if $A\subset V$ is non-empty, define $\tau _A:=(\tau _v)_{v\in A}$. Thus, $\tau _A$ is the vector of infection times of each node in *A*.

In our setting, infection times are observable only for nodes in a nonempty but proper set $\mathcal {O}\subset V$, called the *set of observers*.

In what follows, we write $\tau$ to denote $\tau _\mathcal {O}$. The *source localization problem* we address requires estimating *s* from a single realization of $\tau$. This contrasts with other approaches that assume observers know the nodes from which they were infected.

## Sufficient Statistics for Source Localization

Hereafter, $$\mathcal {O}^c:=V\setminus \mathcal {O}$$.

Several studies have analyzed the placement of observer nodes in a network using various criteria prior to observing infection times (Spinelli et al. 2016; Lokhov and Saad 2017; Li et al. 2019, 2021, 2024). In this section, we argue that only a handful of observer’s infection times are usually needed for estimating the source once their infection times have been observed, as the remaining ones provide only redundant information about it. For this, consider the following equivalence relation between non-observer nodes in *T*: for $u,v \in \mathcal {O}^c$, define

$$\begin{aligned} u \equiv v \text { if and only if } [u,v] \cap \mathcal {O}= \emptyset , \end{aligned}$$

where [*u*, *v*] denotes here the set of vertices on the unique path between *u* and *v* in the tree, including these vertices. The equivalence class of a node $u\in \mathcal {O}^c$ is denoted by ${\textbf {[\hspace{-2.29996pt}[}}u{\textbf {]\hspace{-2.29996pt}]}}$. We note that each equivalence class is a maximal subtree of *T* that does not contain observer nodes. The collection of all equivalence classes is denoted by ${\textbf {[\hspace{-2.29996pt}[}}\mathcal {O}^c{\textbf {]\hspace{-2.29996pt}]}}$.

For each $r\in {\textbf {[\hspace{-2.29996pt}[}}\mathcal {O}^c{\textbf {]\hspace{-2.29996pt}]}}$, the *boundary* of *r*, denoted $\partial r$, is the set of observers adjacent (i.e., connected by an edge) to any node in *r*. Note that each observer can be a neighbor of at most one node in each equivalence class; otherwise, there would be a cycle in *T*. More generally, for $R\subset {\textbf {[\hspace{-2.29996pt}[}}\mathcal {O}^c{\textbf {]\hspace{-2.29996pt}]}}$ we define

$$\begin{aligned} \partial R := \cup _{r\in R} \,\partial r. \end{aligned}$$

Since the equivalence classes partition $\mathcal {O}^c$, the source belongs to a unique equivalence class from which the infection radiates outward. The notion of feasibility, introduced next, formally characterizes the classes from which this outward spread is consistent with the observers’ infection times.

For each $o\in \mathcal {O}$ and $r\in {\textbf {[\hspace{-2.29996pt}[}}\mathcal {O}^c{\textbf {]\hspace{-2.29996pt}]}}$, let $T_{o;r}$ denote the subtree of *T* rooted at *o* containing all nodes in *V* that do not belong to the equivalence class *r*. We denote the vertex set of $T_{o;r}$ as $V_{o;r}$. In particular, $o\in V_{o;r}$ and

1 $$\begin{aligned} V_{o;r}=\big \{v\in V\text { such that }[o,v]\cap r=\emptyset \big \}. \end{aligned}$$

Upon a realization of $\tau$, we say that a class $r\in {\textbf {[\hspace{-2.29996pt}[}}\mathcal {O}^c{\textbf {]\hspace{-2.29996pt}]}}$ is *feasible* when, for all $o\in \partial r$, if $o_1,o_2\in V_{o;r}\cap \mathcal {O}$ are such that $o_1$ is an ancestor of $o_2$ in $T_{o;r}$ then $\tau _{o_1}\le \tau _{o_2}$. If so, $\tau _{o_1}<\tau _{o_2}$ unless $o_1=o_2$ because $\tau _e>0$ for all $e\in E$ almost surely. Intuitively, *r* is feasible when the partial ordering of nodes induced by $T_{o;r}$ is consistent with the infection times of the observers in $V_{o;r}$.

The following result clarifies our terminology.

### Proposition 2.1

With probability one, *s* cannot belong to a non-feasible equivalence class.

### Proof

Suppose that $s\in r$ but *r* is a non-feasible equivalence class; in particular, there exist $o\in \partial r$ and $o_1,o_2\in V_{o;r}$ such that $o_1\in [o,o_2]$ and $[o,o_2]\cap r=\emptyset$ but $\tau _{o_2}<\tau _{o_1}$. Consequently, since we must have $[s,o]\setminus \{o\}\subset r$, $[s,o_2]=[s,o]\cup [o,o_1]\cup [o_1,o_2]$ and the unique path from *s* to $o_2$ must first visit *o*, then $o_1$, and finally $o_2$. Thus, $o_2$ cannot be infected before $o_1$ (i.e., $\tau _{o_2}>\tau _{o_1}$). Since this contradicts the assumption that *r* is non-feasible, we must have $s\notin r$. $\square$

The next result provides a simple characterization of the feasible equivalence classes. We call a set of equivalence classes $R\subset {\textbf {[\hspace{-2.29996pt}[}}\mathcal {O}^c{\textbf {]\hspace{-2.29996pt}]}}$ a *star arrangement* when $\cap _{r\in R}\,\partial r\ne \emptyset$. Any *R* with $|R|=1$ is trivially a star arrangement. On the other hand, if $|R|>1$ is a star arrangement then $\left| \cap _{r\in R}\,\partial r\right| =1$; otherwise, there would be a cycle in *T*.

### Theorem 2.2

With probability one, a class $r\in {\textbf {[\hspace{-2.29996pt}[}}\mathcal {O}^c{\textbf {]\hspace{-2.29996pt}]}}$ is feasible if and only if $\mathop {\mathrm {arg\,min}}\limits _{o\in \mathcal {O}} \tau _o \in \partial r$. In particular, almost surely, at least one feasible equivalence class exists, and the feasible classes form a star arrangement.

In parametric statistics, a *statistic* (i.e., a function of the data that does not depend on any unknown quantity to evaluate) is called *sufficient* to estimate an unknown parameter when the conditional distribution of the data, given the statistic value, does not depend on the parameter (Jem 2022). Sufficient statistics summarize data without losing information relevant to estimating the parameter(s) that controls its probability distribution—in our context, that parameter is the source identity.

The following result characterizes the observers’ infection times that are statistically sufficient for estimating the source.

### Theorem 2.3

**Fig. 1:**
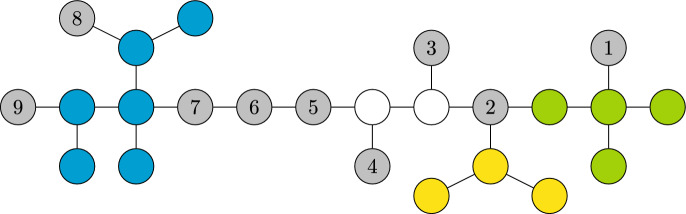
Diagram of an infection tree with observer nodes labeled 1 through 9. It contains four equivalence classes with nodes colored blue, white, yellow, and green. The boundaries of these classes are $\{7,8,9\}$, $\{2,3,5,4\}$, $\{2\}$, and $\{1,2\}$, respectively. The white, yellow, and green classes form a star arrangement (centered at node 2). These three classes are feasible only when observer 2 is the first to become infected; in which case, observers 1 through 5 are sufficient to estimate the source. However, if observer 3 is the first to be infected, only the white class remains feasible, and observers 2 through 5 are sufficient to estimate the source

Let $R\subset {\textbf {[\hspace{-2.29996pt}[}}\mathcal {O}^c{\textbf {]\hspace{-2.29996pt}]}}$ be a star arrangement of classes. If $s\in \cup _{r\in R}\,r$ then $\tau _{\partial R}$ is a sufficient statistic for *s* (See Fig. 1).

Theorems 2.2-2.3 help reduce the complexity of the source localization problem in trees to only consider the infection times of observers in the boundary of (the star arrangement formed by) the equivalence classes that contain the first infected observer. Namely, the $\tau _o$, with $o\in \partial R$, where

2 $$\begin{aligned} R:=\left\{ r\in {\textbf {[\hspace{-2.29996pt}[}}\mathcal {O}^c{\textbf {]\hspace{-2.29996pt}]}}\text { such that }\mathop {\mathrm {arg\,min}}\limits \limits _{w\in \mathcal {O}}\tau _w\,\in \,\partial r\right\} . \end{aligned}$$

In particular, the general source localization problem on trees is reduced to cases where the observers are all leaves—except possibly for a single interior node (the center of a star arrangement). The estimation problem, however, may be substantially more challenging in the latter case.

To clarify the last assertion, suppose the feasible equivalence classes form a star arrangement *R*, with $|R|>1$, centered at $o \in \partial R$. In particular:

3 $$\begin{aligned} \tau _o < \tau _w, \text { for all }w\in \mathcal {O}\setminus \{o\}. \end{aligned}$$

Although Theorem 2.3 asserts that $\tau _{\partial R}$ is sufficient for estimating the source, imposing the above constraint destroys the independence of the edge-delays, because it compels the infection to reach the observer at the center of the arrangement before all others. This not only often renders the distribution of $\tau _{\partial R}$ often intractable, but also places it beyond the scope of existing source estimation methods—including our own in Section 4. A practical way to reframe the source estimation problem in the setting of independent edge-delays is to disregard the knowledge that the center of the arrangement was the first infected node, and to estimate the source as if the network were the subtree induced by the nodes in $R\cup \partial R$.

**Fig. 2:**
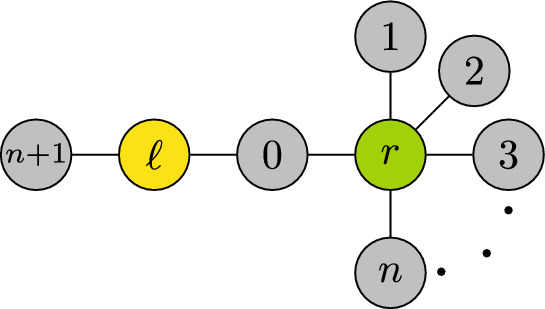
Toy diagram illustrating an infection tree where all the observer nodes, labeled 0 through $(n+1)$, are leaves except for node 0, and the unknown source is either node $\ell$ or node *r*

We emphasize that the statistic $\tau _{\partial R\setminus \{o\}}$ is generally not sufficient for estimating the source in the context of equations (2)-(3). To fix ideas, consider the network in Figure 2 and suppose that $\tau _{\{\ell ,0\}}$ has p.d.f. *f*, whereas $\tau _{\{0,r\}}$ has p.d.f. *g*. Node 0 is the center of the star arrangement of feasible equivalence classes when it is the first to get infected; namely, $\tau _0<\tau _{\min }$ with $\tau _{\min }:=\min _{i=1,\ldots ,n+1}\tau _i$. Conditioning on this event and observing only the infection times in $\tau _{\partial R\setminus \{0\}}$, the conditional p.d.f. of $\tau _0$ depends on the source as follows:

$$\begin{aligned} \mathbb {P}\big (\tau _0=t\,\mid s=\ell ,\,\tau _{\partial R\setminus \{0\}},\,\tau _0<\tau _{\min }\big )&= \frac{f(t)}{\int _0^{\tau _{\min }} f(x)\,dx}; \\ \mathbb {P}\big (\tau _0=t\,\mid s=r,\,\tau _{\partial R\setminus \{0\}},\,\tau _0<\tau _{\min }\big )&= \frac{g(t)}{\int _0^{\tau _{\min }} g(x)\,dx}; \end{aligned}$$

for all $0<t<\tau _{\min }$. In particular, whenever $\tau _{\{\ell ,0\}}$ and $\tau _{\{0,r\}}$ have different distributions (i.e., $\int _0^{\infty }|f(x)-g(x)|\,dx > 0$), the conditional p.d.f.’s above are not guaranteed to be equal. Consequently, $\tau _{\{1,\ldots ,n+1\}}$ is not sufficient for estimating the source, as the conditional distribution of $\tau _0$ may still depend on the source identity.

## Source Identifiability in Trees

In the context of statistical inference, the source is said to be *identifiable* when the distribution of $\tau$ given that $s = v$ is unique for each $v \in V$. Unfortunately, unless the edge-delays are Gaussian (Pinto et al. 2012), explicitly computing the distribution of the vector $\tau$ is non-trivial, especially when the paths connecting observers to an alleged source overlap. Because of this, we use Laplace transforms to characterize the distribution of $\tau$ under each possible source. Importantly, Laplace transforms uniquely determine the distribution of a random vector when they are finite in an open neighborhood of the origin.

We emphasize that an analogous result can be formulated using characteristic functions (O’Connor 2022); however, we choose Laplace transforms because of the non-negative nature of infection times.

The *Laplace transform* of $\tau =(\tau _o)_{o\in \mathcal {O}}$ is the function defined as

4 $$\begin{aligned} \varphi (t):=\mathbb {E}\big (e^{-\langle t,\tau \rangle }\big ),\text { for }t=(t_o)_{o\in \mathcal {O}}\ge 0; \end{aligned}$$

where, hereafter, $t=(t_o)_{o\in \mathcal {O}}\ge 0$ means that $t\in \mathbb {R}_+^\mathcal {O}$. Since the source is unknown in our setting, we denote the above function as $\varphi _v(t)$ when assuming that $s = v$. Namely, for $v\in V\setminus \mathcal {O}$:

$$\begin{aligned} \varphi _v(t) := \mathbb {E}\big (e^{-\langle t,\tau \rangle }\big | s = v\big ), \text { for }t\ge 0. \end{aligned}$$

Our next result provides an explicit formula for the Laplace transform of $\tau$ under each possible source in terms of $\varphi _e$, for $e\in E$, i.e., the Laplace transform of the edge-delays along *T*. To state the result and implement our methods in software, it is convenient to introduce the following matrix with rows indexed by $\mathcal {O}$ and columns indexed by *E*:

$$A_v(o,e) := {\left\{ \begin{array}{ll} 1, & \text {if edge } e \in [v,o]; \\ 0, & \text {otherwise.} \end{array}\right. }$$

### Theorem 3.1

For each $$v\in V\setminus \mathcal {O}$$:

5 $$\begin{aligned} \varphi _v(t) = \prod _{e\in E}\varphi _e\!\!\left( \sum _{o\in \mathcal {O}(e|v)}t_o\right) ,\text { for }t=(t_o)_{o\in \mathcal {O}}\ge 0; \end{aligned}$$

where $\mathcal {O}(e|v):=\big \{o\in \mathcal {O}\text { such that }A_v(o,e)=1\big \}$. In particular, if $t=(t_o)_{o\in \mathcal {O}}$ is represented as a row-vector then

6 $$\begin{aligned} \sum _{o\in \mathcal {O}(e|v)}t_o = (tA_v)(e). \end{aligned}$$

### Remark 1

The elements in the set $\mathcal {O}(e|v)$ are the observers that descend from *e* when the tree *T* is rooted at *v*.

### Proof

If $$s=v$$ then

$$\langle t,\tau \rangle =\sum _{o\in \mathcal {O}}t_o\tau _o =\sum _{o\in \mathcal {O}}t_o\sum _{e\in E}A_v(o,e)\,\tau _e =\sum _{e\in E}\tau _e\sum _{o\in \mathcal {O}}t_o\,A_v(o,e) =\sum _{e\in E} (tA_v)(e)\,\tau _e.$$

In particular, since $\tau _e$, with $e\in E$, are independent:

$$\varphi _v(t) =\prod _{e\in E}\mathbb {E}\left( e^{-(tA_v)(e)\,\tau _e}\right) =\prod _{e\in E}\varphi _e\Big ((tA_v)(e)\Big ).$$

Since $(tA_v)(e)=\sum _{o\in \mathcal {O}(e|v)}t_o$, the result follows. $\square$

**Fig. 3:**
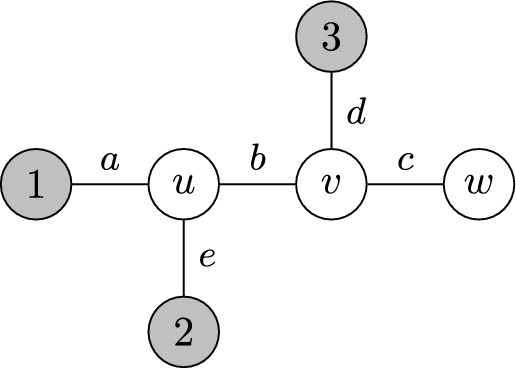
Example of an infection tree with observers labeled 1 to 3 (colored gray), non-observer nodes labeled *u*, *v*, and *w*, and edges set $\{a,b,c,d,e\}$

To fix ideas about our last result, consider the infection tree in Figure 3. Due to equation (5), the Laplace transform of $\tau = (\tau _1,\tau _2,\tau _3)$ evaluated at $t = (t_1,t_2,t_3)$, depending on the identity of the source, is given by

$$\begin{aligned} \varphi _u(t)&= \varphi _a(t_1)\cdot \varphi _b(t_3)\cdot \varphi _d(t_3)\cdot \varphi _e(t_2);\\ \varphi _v(t)&= \varphi _a(t_1)\cdot \varphi _b(t_1+t_2)\cdot \varphi _d(t_3)\cdot \varphi _e(t_2);\\ \varphi _w(t)&= \varphi _a(t_1)\cdot \varphi _b(t_1+t_2)\cdot \varphi _c(t_1+t_2+t_3)\cdot \varphi _d(t_3)\cdot \varphi _e(t_2). \end{aligned}$$

Table 1 displays the Laplace transforms of the edge-delay distributions we use to evaluate our results.

**Table 1:** Some continuous distributions on the positive real line and their corresponding Laplace transforms in terms of their parameters. By PosNormal we refer to a Gaussian distribution with mean $\mu$ and variance $\sigma ^2$, conditioned to be nonnegative. In the table, $\Phi$ is the cumulative distribution function of a standard normal, respectively. The AbsCauchy distribution refers to the absolute value of a Cauchy random variable with location and scale parameters 0 and $\sigma$, respectively. In the table, $\text {Ci}$ and $\text {Si}$ are the cosine and sine integrals, respectively

Distribution	Parameters	Laplace Transform
$\text {Exponential}(\lambda )$	$\lambda >0$	$\frac{\lambda }{\lambda +t}$
$\text {PosNormal}(\mu ,\sigma ^2)$	$\mu \ge 0,\,\sigma >0$	$\frac{\Phi \left( (\mu /\sigma )-\sigma t\right) }{\Phi \left( \mu /\sigma \right) }\,e^{-\mu t + \frac{\sigma ^2t^2}{2}}$
$\text {Uniform}(a,b)$	$0\le a< b <+\infty$	$\frac{e^{-at}-e^{-bt}}{(b-a)\,t}$
$\text {AbsCauchy}(\sigma )$	$\sigma > 0$	$\frac{1}{\pi }\left( 2\,\text {Ci}(t\sigma )\sin (t\sigma )+\cos (t\sigma )\left( \pi -2\,\text {Si}(t\sigma )\right) \right)$

## Laplace-based Source Localization

Classical statistical inference techniques for point estimation aim to minimize the mean square error between a statistic and an unknown parameter—implicitly restricting the statistics of interest to those with finite second (and consequently first) moments. Other methods, such as maximum likelihood estimation, rely on explicit formulas for the joint distribution of the data.

In the context of observer-based source localization, however, the observers’ infection times often lack explicit joint density functions or finite second moments. In situations analogous to this, some point estimation methods have exploited characteristic functions to estimate parameters (Feuerverger and McDunnough 1981a, b; Madan and Seneta 1987; Elgin 2011; Berry and Kleiber 2023). The central idea of these methods is that the empirical characteristic function of the data converges to the characteristic function of its distribution over compact sets as the sample size increases. In particular, the parameters of the unknown distribution can be estimated by fitting the characteristic function to its empirical counterpart.

In this section, we adapt the latter methodology to estimate the source of infection in a tree by working with Laplace transforms instead of characteristic functions. This choice is appropriate not only because infection times are non-negative, but also due to the explicit formula for the Laplace transform of the observers’ infection times given in Theorem 3.1.

The *empirical Laplace transform* of the vector of observer’s infection time $\tau$ is the function $\hat{\varphi }:\mathbb {R}_+^\mathcal {O}\rightarrow [0,1]$ defined as

7 $$\begin{aligned} \hat{\varphi }(t) := e^{-\langle t,\tau \rangle },\text { for }t=(t_o)_{o\in \mathcal {O}}\ge 0. \end{aligned}$$

For each *t* like above, $\hat{\varphi }(t)$ is an unbiased estimator of $\varphi (t)$.

In the traditional approach, one selects a grid of values $t_1, \ldots , t_n\in \mathbb {R}_+^\mathcal {O}$ and estimates the source by minimizing over $v \in \mathcal {O}^c$ the quantity

$$\begin{aligned} \sum _{j=1}^n\big (\hat{\varphi }(t_j)-\varphi _v(t_j)\big )^2. \end{aligned}$$

This approach, however, has the disadvantage of not being scale-invariant: *if the units of time are changed by a constant factor (e.g., measuring time in weeks instead of days), the source estimator may also change*.

To address the scale-invariance issue, we fix *p* such that $2\le p\le +\infty$ and instead aim to solve the optimization problem

8 $$\begin{aligned} \min _{v\in V\setminus \mathcal {O}}\Vert \hat{\varphi }-\varphi _v\Vert _p, \end{aligned}$$

where $\Vert \cdot \Vert _p$ denotes the $L^p$-norm on $\mathbb {R}_+^\mathcal {O}$ with respect to the Lebesgue measure. (Weighted $L^p$-norms may also be used, provided the weighting function is homogeneous to keep the source estimator scale-invariant.)

Since $\hat{\varphi }$ is almost surely a linear combination of functions in $L^p$, $\hat{\varphi }\in L^p$. On the other hand, the Laplace transform is a continuous linear operator from $L^q$ to $L^p$, where $q:= p / (p - 1)\in [1,2]$ is interpreted as 1 when $p=+\infty$ (Peachey 2022). In particular, if the probability density function of $\tau$ is in $L^q$, then the objective function above is finite for each $v\in V\setminus \mathcal {O}$. Unfortunately, however, for $2 \le p < +\infty$, computing the $L^p$-norm in (8) is computationally demanding, particularly in high dimensions. Moreover, since in general we can only assert that the p.d.f. of $\tau$ lies in $L^1$, selecting $p = +\infty$ is a natural choice. Accordingly, we propose estimating the source by solving the following optimization problem:

9 $$\begin{aligned} \hat{s} :=\mathop {\mathrm {arg\,min}}\limits _{v\in V\setminus \mathcal {O}}\Vert \hat{\varphi }-\varphi _v\Vert _\infty =\mathop {\mathrm {arg\,min}}\limits _{v\in V\setminus \mathcal {O}}\,\sup _{t\in \mathbb {R}^{|\mathcal {O}|}_+}\left| \hat{\varphi }(t)-\varphi _v(t)\right| . \end{aligned}$$

We call this the *source-hat estimator*. In practice, we solve the above optimization problem using a modification of the Nelder-Mead method (Gao and Han 2012).

### Alternative Source Estimator

We address now how to improve the source estimator in (9). A drawback of the alternative estimator is its reliance on explicit expressions for certain conditional Laplace transforms. In this regard, the main result of this section (Theorem 4.2) provides such a formula, albeit in terms of convolution operators, which may still be challenging to compute explicitly in practice. Nonetheless, it enables the derivation of explicit expressions for conditional Laplace transforms in networks with Exponential delays.

Guided by Theorem 7.1 in the appendix, we can estimate the conditional Laplace transform of $\tau =(\tau _o)_{o\in \mathcal {O}}$ given that $s=v$ by

10 $$\begin{aligned} \check{\varphi }_v(t)&:=\frac{|\mathcal {O}|-1}{2|\mathcal {O}|-1}e^{-\langle t,\tau \rangle }+\frac{1}{2|\mathcal {O}|-1}\sum _{o\in \mathcal {O}}\varphi _v(t|\tau _o), \end{aligned}$$

where

11 $$\begin{aligned} \varphi _v(t|\tau _o):=\mathbb {E}\left( \left. e^{-\langle t, \tau \rangle }\right| s=v,\tau _o\right) . \end{aligned}$$

This leads us to the following alternative source estimator:

12 $$\begin{aligned} \check{s} :=\mathop {\mathrm {arg\,min}}\limits _{v\in V\setminus \mathcal {O}}\Vert \check{\varphi }_v-\varphi _v\Vert _\infty =\mathop {\mathrm {arg\,min}}\limits _{v\in V\setminus \mathcal {O}}\,\sup _{t\in \mathbb {R}^{|\mathcal {O}|}_+}\left| \check{\varphi }_v(t)-\varphi _v(t)\right| . \end{aligned}$$

We call this the *source-check estimator*, which we also compute in practice using the Nelder-Mead method (Gao and Han 2012).

Importantly, while $\hat{\varphi }$ and $\check{\varphi }_v$ are both unbiased estimators of $\varphi _v$ when $s = v$, the variance of the latter can never exceed that of the former. In particular, source estimation based on the optimization in (12) should be preferred over that in (9)—provided that $\check{\varphi }_v$ is computationally tractable for each $v\in V\setminus \mathcal {O}$.

The conditional Laplace transform in (11) can be made more explicit by following a similar line of reasoning to that used in the proof of Theorem 3.1, as stated next (proof omitted).

#### Corollary 4.1

For all $v\in V\setminus \mathcal {O}$ and $o\in \mathcal {O}$:

$$\begin{aligned} \varphi _v(t|\tau _o)&=\mathbb {E}\left( \left. \prod _{e\in [v,o]}e^{-\tau _e\cdot \sum \limits _{o'\in \mathcal {O}(e|v)}\!\!\!\!t_{o'}}\right| s=v,\tau _o\right) \cdot \prod _{e\notin [v,o]}\varphi _e\!\!\left( \sum _{o\in \mathcal {O}(e|v)}\!\!\!\!t_o\right) . \end{aligned}$$

For instance, for the infection tree in Figure 3, the corollary implies that

$$\begin{aligned} \varphi _u(t|\tau _3)&= \varphi _a(t_1)\cdot \varphi _e(t_2)\cdot e^{-t_3\tau _3};\\ \varphi _v(t|\tau _3)&= \varphi _a(t_1)\cdot \varphi _b(t_1+t_3)\cdot \varphi _e(t_2)\cdot e^{-t_3\tau _3};\\ \varphi _w(t|\tau _3)&=\varphi _a(t_1)\cdot \varphi _b(t_1+t_2)\cdot \varphi _e(t_2)\cdot \mathbb {E}\left( \left. e^{-(t_1+t_2+t_3)\tau _c-t_3\tau _d}\right| s=w,\tau _c+\tau _d\right) ; \end{aligned}$$

where we used $\tau _3 = (\tau _b + \tau _d)$ when $s=u$ in the top identity, and $\tau _3 = (\tau _c + \tau _d)$ when $s=w$ in the bottom identity.

The explicit formulas in the first two examples above are uncommon, whereas the third is a more typical albeit simple example of the type of conditional expectations required to compute conditional Laplace transforms of the form given in Corollary 4.1. The following result provides a general formula for conditional Laplace transforms of this type, which rely on the convolution operator.

For each $c\ge 0$, define the the $L^1$-endomorphism:

$$\begin{aligned} (\mathcal {L}_cf)(x):=e^{-c x}f(x),\,x\ge 0. \end{aligned}$$

#### Theorem 4.2

Let $k\ge 2$ be an integer. If $c_1,\ldots ,c_k\ge 0$ are given real numbers, and $\tau _1,\ldots ,\tau _k\ge 0$ are independent continuous random variables with p.d.f.’s $f_1,\ldots ,f_k$, respectively, then

13 $$\begin{aligned} \mathbb {E}\left( \left. e^{-\sum \limits _{i=1}^kc_i\tau _i}\right| \sum _{i=1}^k\tau _i=t\right) =\frac{(\mathcal {L}_{c_1}f_1*\cdots *\mathcal {L}_{c_k}f_k)(t)}{(f_1*\cdots *f_k)(t)},\,\text { for all }t\in \mathbb {R}_+^k. \end{aligned}$$

We can provide an explicit formula for equation (13) when $\tau _1,\ldots ,\tau _k$ i.i.d. exponential random variables.

#### Corollary 4.3

Let $k\ge 2$ be an integer. If $c_1,\ldots ,c_k\ge 0$ are constants, and $\tau _1,\ldots ,\tau _k$ i.i.d. $\text {Exponential}(\lambda )$ random variables, then

14 $$\begin{aligned} \mathbb {E}\left( \left. e^{-\sum \limits _{i=1}^kc_i\tau _i}\right| \sum _{i=1}^k\tau _i=t\right) =g(t)\,t^{k-1}e^{-\lambda t}\,(k-1)!\cdot \prod _{i=1}^k\frac{1}{\lambda +c_i},\,\text { for all }t\in \mathbb {R}_+^k; \end{aligned}$$

where *g*(*t*) is the p.d.f. of a sum of independent exponential random variables with rates $(\lambda +c_1),\ldots ,(\lambda +c_k)$, respectively.

#### Proof

Let $f_i$ and $\varphi _i$ be the p.d.f. and Laplace transform of $\tau _i$, respectively. Let $X_1,\ldots ,X_k$ be independent random variables such that, for each $1\le i\le k$, $X_i$ has p.d.f. $g_i:=\mathcal {L}_{c_i}f_i/\varphi _i(c_i)$. Then

$$\varphi _{X_i}(t)=\int _0^\infty \frac{e^{-(t+c_i)x}f_i(x)}{\varphi _i(c_i)}\,dx =\frac{\varphi _i(t+c_i)}{\varphi _i(c_i)}=\frac{\lambda +c_i}{\lambda +c_i+t}.$$

In particular, $X_i\sim \text {Exponential}(\lambda +c_i)$, and $g:=(g_1*\cdots *g_k)$ is the p.d.f. $\sum _{i=1}^kX_i$. The corollary follows from equation (17) in the proof of Theorem 4.2 in the appendix. $\square$

#### Remark 2

If $X_1,\ldots ,X_k$ are independent exponentials with rates $\lambda _1,\ldots ,\lambda _k>0$, respectively, then $\sum _{i=1}^k X_i$ is said to have a *hypoexponential* (a.k.a. generalized Erlang distribution) distribution. In the special case when $\lambda _i\ne \lambda _j$ for all $i\ne j$, the p.d.f. of this distribution is

$$\begin{aligned} g(t):=\sum _{i=1}^k \lambda _i\,e^{-\lambda _i\,t}\cdot \prod _{j\ne i}\frac{\lambda _j}{\lambda _j-\lambda _i},\text { for all }t\in \mathbb {R}_+. \end{aligned}$$

Hypoexponential distributions are particular instances of the so-called *continuous phase type distribution*. In particular, if two or more of the rates $\lambda _1, \ldots , \lambda _k$ are repeated, the distribution of $\sum _{i=1}^k X_i$ is of phase type; in this case, its c.d.f. and p.d.f. can be computed using matrix exponentiation (Legros and Jouini 2015).

## Source Localization Performance and Application

In this section, we evaluate the performance of our source localization methods using synthetic data on usually random networks (Sections 5.1-5.3) as well as synthetic data on a real river network (Section 5.2).

The computation of infinity norms in (9) and (12) was performed using the multidimensional Nelder-Mead optimization method as implemented in Gao and Han (2012). This method was chosen for its effective handling of constrained optimizations, speed, and robustness.

Our tests in Section 5.1 use the source estimator in equation (9), whereas those in Section 5.3 use the one in (12). In both sections, the observers are chosen as a subset of the leaves to avoid the difficulties discussed at the end of Section 2. This is not the case for the data in Section 5.2, however, the localization problem can be reduced to the previous case using the tools in Section 2.

We consider synthetic infection networks with i.i.d. edge-delays drawn from the following distributions (see Table 1):

- $$\text {PosNormal}(1,\sigma ^2)$$, with $$\sigma ^2 = 1/16,\ 1/4,\ 1$$
- $$\text {Exponential}(1)$$;
- $$\text {Uniform}(0,2)$$;
- $$\text {AbsCauchy}(1)$$.

For the first three of these distributions, the edge-distance between an observer and the source is proportional to the observer’s expected infection time—and exactly equal to it for the middle two distributions. This does not hold for the fourth distribution, which has infinite moments of all orders. However, we selected $\sigma = 1$ because it gives a distribution with median 1. We note that, because our methods are scale invariant, their performance under i.i.d. Exponential delays, or Uniform delays anchored at 0, does not depend on the parameters of these distributions.

Each of the above distributions reflects characteristics of practical or theoretical interest. Indeed, when the variance is small relative to the mean, the positive Normal distribution models delays resulting from the aggregation of multiple independent and short-lived delays due to the various ways in which the standard Central Limit Theorem may emerge. The Exponential is well-suited for modeling Markovian (i.e., memoryless) delays, while the Uniform distribution serves as a paradigm for high-entropy delays; in particular, Uniform delays offer minimal information about the location of a source. Finally, due to the heavy tail of the Cauchy distribution, anomalously high edge-delays are likely to occur along long paths connecting a node to the source, making localization particularly challenging.

### Hat-estimator Performance on Synthetic Networks

In this section, we test the hat-estimator as defined in equation (9).

**Fig. 4:**
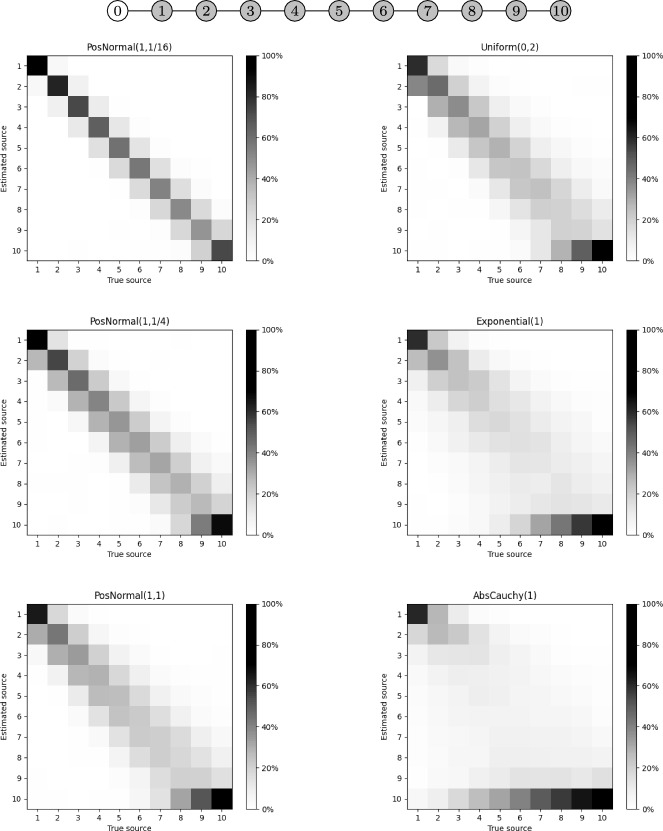
Diagram of a path infection network with a single observer (top row) and confusion matrices for source localization based on the $\hat{s}$ estimator when using i.i.d. PosNormal (left column), Uniform (top right column), Exponential (center right column), and AbsCauchy (bottom right column) edge-delay distributions. Each of these was run with 1,000 samples for each possible true source. The darker the shading along the diagonals and the lighter the shading off them, the better the source localization performance

To begin, we consider a path tree with an observer at its left end (labeled 0) and ten potential sources (labeled $1,\ldots ,10$) to its right—see the top of Figure 4. This simple network is well-suited for testing our methodology because—except under AbsCauchy edge-delays—the variance of the observer’s infection time increases proportionally with its edge-distance from the true source.

As seen in Figure 4, the confusion matrices corresponding to the PosNormal distributions with $\sigma =1/4,1/2$ are mostly concentrated around the diagonal, indicating that $\hat{s}$ often correctly identifies *s* or a nearby node. However, as expected, the larger the variance of the PosNormal distribution, the less accurate $\hat{s}$ becomes at identifying the true source, particularly as the observer moves farther from it. In fact, $\hat{s}$ performs slightly better under the Uniform distribution with a standard deviation of $1/\sqrt{3}\approx 0.577$ than under the PosNormal distribution with parameter $\sigma ^2=1$, which has an approximate standard deviation of 0.794.

In contrast, the estimator’s performance deteriorates dramatically for the Exponential and the Absolute Cauchy distribution. The poor performance under the Exponential distribution may be attributed to its unit variance, which is larger than that of any of the PosNormal and Uniform edge-delay distributions considered. On the other hand, due to the heavy tails of the Cauchy distribution, the estimator’s performance deteriorates rapidly as the source moves farther from the observer.

Across all these tests, node 10 is the most likely to be incorrectly estimated as the source. While this is partly due to the increased variance associated with its location furthest from the true source, it primarily occurs because node 10 is a terminal node. In fact, if an edge-delay between the observer and the source is unexpectedly large (an event that becomes more likely as the distance between them increases), $\hat{s}$ defaults to selecting the node most likely to produce extended infection times.

**Fig. 5:**
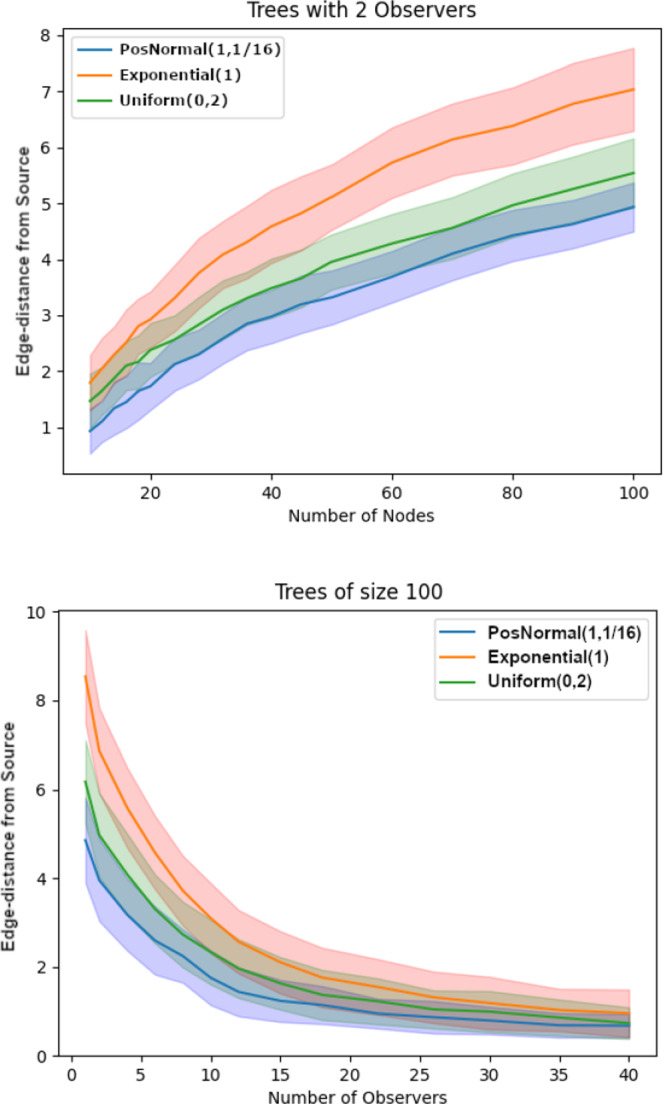
Top: Average edge-distance (i.e., number of edges) between $\hat{s}$ and *s* in infection trees with only 2 observers, as the size of randomly generated trees increases. Each tree size had 1,000 samples. Bottom: Average edge-distance in randomly generated trees with 100 nodes, as the number of observers increases. Each number of observers had 1,000 samples. In all the plots, the shaded bands represent ± one standard deviation from the mean

To evaluate the effectiveness of the $\hat{s}$ estimator in more general infection networks and investigate its accuracy and sensitivity to the structural characteristics of the network, we conducted two types of experiments on random trees varying in size and number of observers.

These random trees were selected uniformly at random from the set of all trees with *n* nodes. This was done by generating Prüfer sequences (Prüfer 1918) uniformly at random and then building the related trees. (Prüfer sequences of length $(n-2)$ are in bijection with trees containing *n* nodes.) All observers were selected to lie on the leaves to avoid any issues with the star arrangement configurations discussed earlier.

In the first type of experiment, we fixed the number of observers at 2 while increasing the network size, which resulted in an observer density ranging from 20% to 2%. As shown on the left of Figure 5, the average edge-distance between $\hat{s}$ and *s* increased sub-linearly while the standard deviation was approximately within the range of a single network edge. In contrast, in the second type of experiment, we fixed the network size at 100 nodes and increased the observer density from 1% to 40%. As shown on the right of Figure 5, the average edge-distance between $\hat{s}$ and *s* decreased sub-linearly, while the standard deviation again remained within the range of a single edge.

**Fig. 6:**
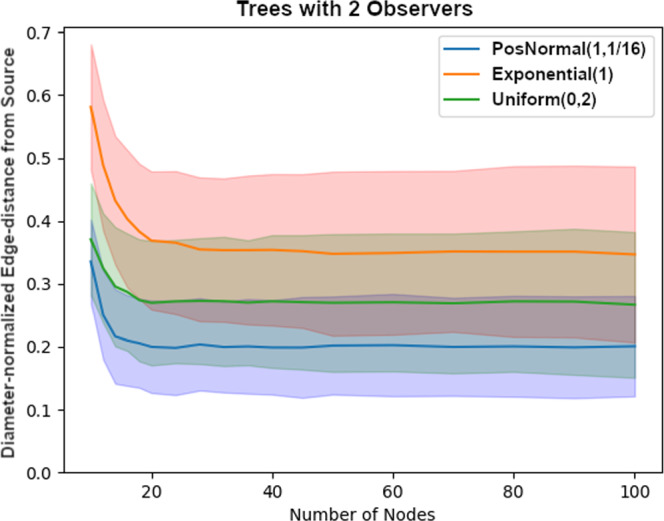
Performance of the method normalized by the diameter of the tree in a tree with 2 observers vs. the size of the tree for uniformly at random generated trees with i.i.d. normal, exponential, and uniform edge-delay distributions. Each node-size was run with 1,000 samples

Next, we explored how does the edge-distance between *s* and $\hat{s}$ compare to the diameter of the tree (i.e., largest edge-distance between a pair of nodes in the network). As seen on Figure 6, the average diameter-normalized edge-distance between *s* and $\hat{s}$ becomes essentially constant for each of the three edge-delay distributions tested as the observer density decreases by holding the number of observers fixed at 2. This is somewhat expected because average edge-distance between the observers and a randomly placed source should grow proportionally with the network size.

### Hat-estimator Performance in a River Network

**Fig. 7:**
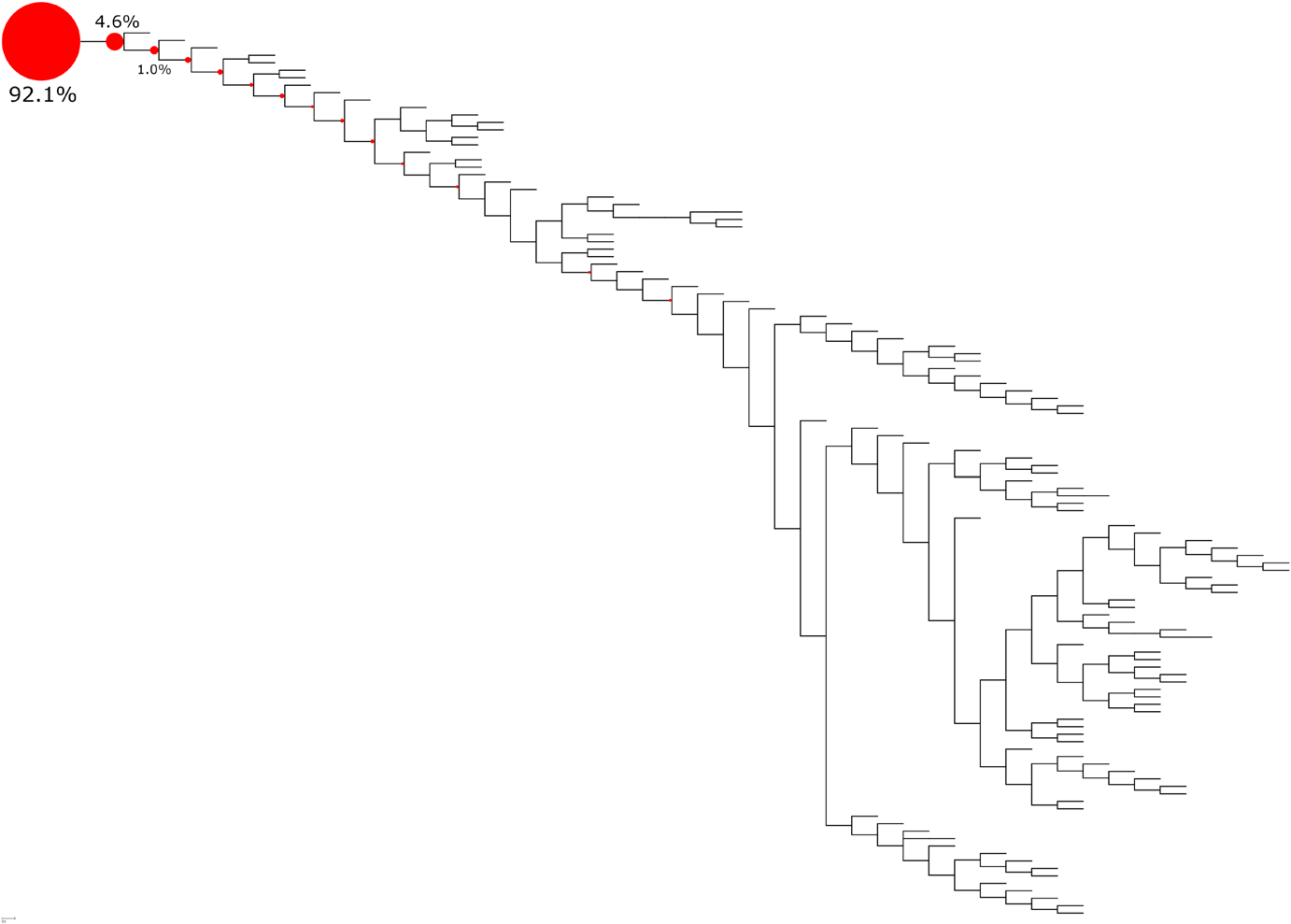
Heatmap of the empirical probability that each node in the river network is identified as the source when the infection originates at the root of the periodogram. Larger nodes correspond to those more frequently predicted as the source by the $\hat{s}$ estimator across 1,000 trials. In each trial, three nodes downstream from the root were selected uniformly at random as observers

In real-world settings, infection trees are rarely uniformly distributed over the set of all possible trees and often exhibit structural features shaped by factors such as geography, contact patterns, or transmission dynamics.

To assess the performance of the hat-estimator in a more realistic scenario, we consider an infection network from a cholera outbreak in the KwaZulu-Natal province of South Africa in the year 2000. This epidemic was caused by a strain of *Vibrio cholerae*, which typically spreads through aquatic environments—in this case, along the Thukela River basin. Because the infection followed a river system, the resulting network naturally forms a directed tree-like structure.

This network is considered in the context of source localization by Pinto et al. (2012), where edge-delays were modeled using Normal distributions with parameters estimated by Bertuzzo et al. (2010, 2008), who modeled infection propagation with a system of differential equations.

Here, in each trial, the source was set to the root of the network, and three observers were selected uniformly at random, excluding the root. For the edge-delays, we reused the parameters in Pinto et al. (2012), but assumed the delays follow Positive Normal distributions. This adjustment has a negligible impact on the original model, since the probability mass below zero is marginal.

We emphasize that the directional flow of water along the river is still compatible with our methods, although they were originally developed for undirected trees. In fact, because the directed nature of the edges rules out candidate sources downstream of the infected observers, in each trial, the source can still be estimated by reducing the river network to the subtree of nodes upstream of the three observers, as if the network were undirected.

As seen in Figure 7, our method identifies the true source in almost all trials. Moreover, with only 3 observers placed at random among the 246 nodes in the river basin, the estimator finds the true source 92.1% of the time, while approximately 98% of the estimates fall within the three nodes nearest (in terms of edge-distance) to the true source. These same nodes are also the most frequently identified as the source and represent only about 1% of all nodes where the infection could have originated.

### Check-estimator Performance under Markovian Delays

In this section, we test the check-estimator as defined in equation (12) and compare its performance to that of the hat-estimator of the infection source. We recall that the former estimator relies on formulas for conditional Laplace transforms, which we determined explicitly only for exponential delays. Nevertheless, this class of edge-delay distributions may be well-suited to settings in which information is transmitted through a network in a reasonably memoryless manner.

**Fig. 8:**
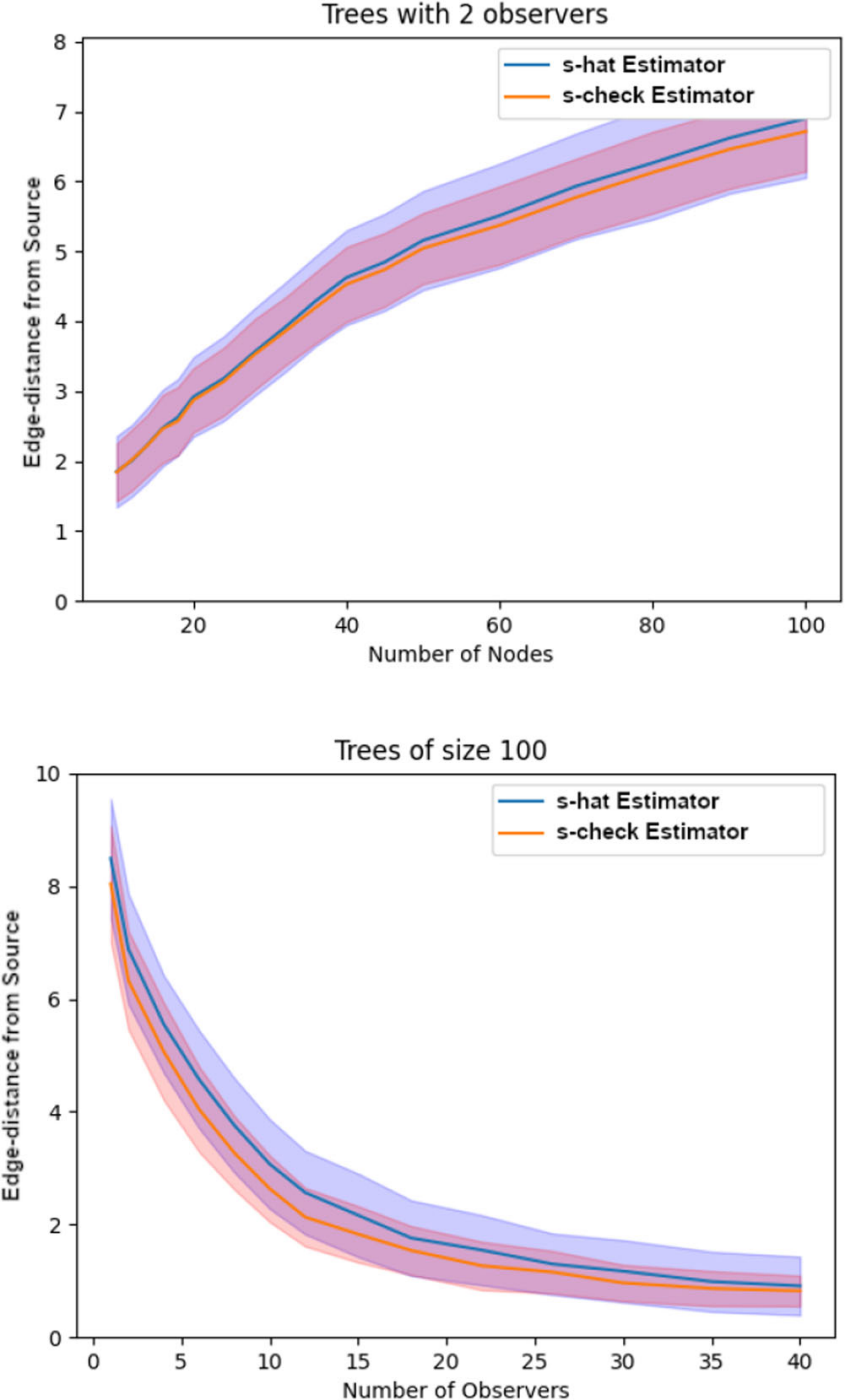
Top: Average edge-distance between $\hat{s}$ and *s*, and $\check{s}$ and *s*, in infection trees with exponential delays and only 2 observers, as the size of randomly generated trees increases. Bottom: Average edge-distance between $\hat{s}$, $\check{s}$ and *s* in randomly generated infection trees of fixed size with exponential delays, as the number of observers increases. In both plots, the shaded bands represent ± one standard deviation from the mean, estimated from 1,000 simulations at each value along the abscissa

As seen in Figure 8, the $\check{s}$-estimator performs on average marginally better than the $\hat{s}$-estimator in terms of edge-distance to the true source, in both low- and high-observer-density scenarios. However, as seen in the same plots, the standard deviation of the edge-distance between $\check{s}$ and *s* is often markedly lower than that of $\hat{s}$. This feature is consistent with the variance reduction technique (see Section 7.3) that motivated the definition of the source-check estimator in (12). Thus, when the conditional Laplace transforms of the form in Theorem 4.2 can be computed explicitly, the $\check{s}$ estimator should be preferred over its precursor.

Altogether, the simulations in this and the preceding sections make a compelling case for our Laplace-derived estimators for source localization in SI networks with a tree structure. In the next section, however, we show that this structure is too restrictive for source estimation in networks with more complex topologies.

## Concluding Remarks

We have studied theoretical aspects of identifiability and complexity in estimating the source of infection in undirected tree networks, where only a subset of nodes (the observers) report their infection times. Our methods rely on the joint Laplace transform of these times rather than on their joint probability density, which is often intractable.

We have assumed that each observer reported only a single infection time. This is realistic at the onset of biological epidemics, but it makes accurate source estimation considerably more difficult. Nevertheless, our methods can be directly extended to situations with multiple vectors of observer infection times, for example, when a hidden bad actor repeatedly spreads misinformation on a social network. In such cases, the variance of the empirical Laplace transforms of the observers’ infection times can be reduced via averaging in equations (7) and (10).

Our methods are scale-invariant and apply to any contagion model between neighboring nodes, provided that the transmission delays of infection along edges are (probabilistically) independent and admit explicit Laplace transforms. In particular, they cover a wide range of edge-delay models, including mixed ones, beyond the well-studied case of Gaussian delays.

We tested our methods across a wide range of random tree networks and i.i.d. edge-delay models, varying the network sizes and proportion of observers. As expected, experiments on a linear network show that the performance of our first method (the source-hat estimator) degrades as the observer moves farther from the source due to the increased edge-distance (a proxy for the variance of infection times in i.i.d. edge-delay models). On average, however, the edge-distance between the estimator and the true source scaled sub-linearly with the observer density—rising as the density decreased and falling as it increased. The performance improved with our second method (the source-check estimator), which we evaluated on networks with exponential (memoryless) edge-delays.

## Technical Proofs and Auxiliary Results

### Proof of Theorem 2.2

Since *T* is connected, for any equivalence class *r* and $o_1\in \mathcal {O}$, there exists $o_2\in \partial r$ such that $o_1\in V_{o_2;r}$. But, if *r* is feasible, then for all $o\in \partial r$ and $\omega \in V_{o,r}$, we have $\tau _o\le \tau _\omega$, with equality only if $\omega =o$. Hence, $\tau _o$, for $o\in \mathcal {O}$, must be minimized at some $o\in \partial r$.

To show the converse, suppose that $\omega = \mathop {\mathrm {arg\,min}}\limits _{o\in \mathcal {O}} \tau _o \in \partial r$ but that *r* is not feasible. Let $o\in \partial r$ and $o_1,o_2\in V_{o,r}$ be such that $o_2$ descends from $o_1$ in $T_{o,r}$ but $\tau _{o_2} < \tau _{o_1}$.

If $s\notin V_{o;r}$, the only way the infection can reach $o_2$ is by first infecting $o_1$, contradicting the assumption that $\tau _{o_1} > \tau _{o_2}$. Hence, $s\in V_{o;r}$. However, to infect $\omega$, the infection must first reach *o*, which is only possible if $o = \omega$; otherwise, $\omega$ could not have the smallest infection time among the observers.

Let $s\wedge o_2$ be the least common ancestor of *s* and $o_2$ in $T_{o;r} \,(=T_{\omega ;r})$. In particular, we have $s\wedge o_2\in [o_2,o]=[o_2,o_1]\cup [o_1,o]$. Since $s\wedge o_2\in [o_1,o]$ is not possible because $\tau _{o_2}<\tau _{o_1}$, it must be the case that $s\wedge o_2\in [o_2,o_1]\setminus \{o_1\}$. But then $\tau _{s\wedge o_2}<\tau _{o_1}\le \tau _o=\tau _\omega$, which is again not possible. Consequently, *r* must be feasible, completing the proof of the theorem.

### Proof of Theorem 2.3

Let $T_R=(V_R,E_R)$ be the subgraph of *T* with vertex set $V_R=\cup _{r\in R}(r\cup \partial r)$. Because *R* is a star arrangement, $T_R$ is connected; hence it is a subtree of *T*. In particular, since $s\in V_R$, there is a unique path connecting *s* to each vertex in $V_R$, and this path is contained in $T_R$. Hence, for each $o\in \partial R\,(\subset V_R)$, $[s,o]\subset E_R$, and the distribution of $\tau _o=\sum _{e\in [s,o]}\tau _e$ is solely determined by the edge-delays in $E_R$. Consequently, the distribution of $\tau _{\partial R}$ depends only on the delays $\tau _e$, with $e\in E_R$.

On the other hand, because $s\in V_R$ and *T* is connected, for each $\omega \in \mathcal {O}\setminus V_R$, there is a unique path from $\omega$ to *s*, and this path must enter $V_R$ at some observer $o\in \partial R$. In particular, $[\omega ,s]=[\omega ,o]\cup [o,s]$, hence $\tau _w=\tau _o+\sum _{e\in [o,\omega ]}\tau _e$. But each $e\in [o,\omega ]$ lies outside the subtree $T_R$. As a result, $[o,\omega ]\subset E\setminus E_R$ and $(\tau _{\omega } - \tau _o)=\sum _{e\in [o,\omega ]}\tau _e$ is independent of $\tau _o$, because the latter is only determined by the delays $\tau _e$ with $e\in E_R$, and the distribution of $(\tau _{\omega } - \tau _o)$ remains the same regardless of the identity of the source node in $V_R$; which shows the theorem.

### Improving Single Multidimensional-Sample Estimation

Let $X=(X_1,\ldots ,X_d)$ be a random vector and $F:\mathbb {R}^d\rightarrow \mathbb {R}$ a given function. Define $\theta =\mathbb {E}\big (F\big )$; in particular, $F:=F(X)$ is an unbiased estimator of $\theta$.

Next, we show how to derive from *F* another unbiased estimator of $\theta$ with smaller variance, provided that—on average—the conditional variance of *F* given any $X_i$ is comparable to that of *F* without conditioning. The modified estimator resembles the Hájek projection of *F*(*X*) (Van Der and Vaart 1998), although the latter would assume that $X_1, \ldots , X_d$ are independent.

#### Theorem 7.1

Assume that $\mathbb {E}(F^2)<+\infty$ and $\mathbb {E}(F)=\theta$. Define

15 $$\begin{aligned} G:=\frac{d-1}{2d-1}F+\frac{1}{2d-1}\sum _{i=1}^d\mathbb {E}(F|X_i);\text { in particular},\mathbb {E}(G)=\theta . \end{aligned}$$

If $\alpha \ge 0$ is such that $\mathbb {E}\left( \frac{1}{d}\sum \limits _{i=1}^d\mathbb {V}(F|X_i)\right) \ge \alpha \cdot \mathbb {V}(F)$, then

16 $$\begin{aligned} \mathbb {V}(G) \le \left( 1-\frac{\alpha \,d}{2d-1}\right) \mathbb {V}(F). \end{aligned}$$

#### Remark 3

$\alpha \le 1$ because $\mathbb {E}\big (\mathbb {V}(F|X_i)\big )\le \mathbb {V}(F)$ for each *i*. In particular, $\mathbb {V}(G)\le \mathbb {V}(F)$.

#### Proof

Consider $0\le \lambda \le 1$ to be selected later, and define

$$\begin{aligned} G:=\lambda F+\frac{1-\lambda }{d}\sum _{i=1}^d\mathbb {E}(F|X_i). \end{aligned}$$

The statistic in (15) corresponds to $\lambda =(d-1)/(2d-1)$, which we will see is optimal for the inequality in (16).

For the sake of a simpler notation, let $E_i:=\mathbb {E}(F|X_i)$ and $V_i:=\mathbb {V}\big (F|X_i)$. Then

$$\begin{aligned} \mathbb {V}(G)&=\lambda ^2\mathbb {V}(F)+\frac{2\lambda (1-\lambda )}{d}\sum _{i=1}^d\text {cov}\big (F,E_i\big )+\frac{(1-\lambda )^2}{d^2}\sum _{i,j=1}^d\text {cov}\big (E_i,E_j\big ). \end{aligned}$$

But note that $\mathbb {E}(E_i)=\mathbb {E}(F)$; in particular

$$\begin{aligned} \text {cov}\big (F,E_i\big )&= \mathbb {E}(F\cdot E_i)-\mathbb {E}(F)\cdot \mathbb {E}(E_i) \\&= \mathbb {E}\big (\mathbb {E}(F\cdot E_i|X_i)\big )-\big [\mathbb {E}(E_i)\big ]^2 \\&= \mathbb {E}\big (E_i^2\big )-\big [\mathbb {E}(E_i)\big ]^2 \\&= \mathbb {V}(E_i). \end{aligned}$$

On the other hand, $\text {cov}(E_i,E_j)=\mathbb {V}(E_i)$ when $i=j$. As a result:

$$\begin{aligned} \mathbb {V}(G)&=\lambda ^2\mathbb {V}(F)\!+\!\left( \frac{2\lambda (1-\lambda )}{d}\!+\!\frac{(1-\lambda )^2}{d^2}\right) \sum _{i=1}^d\mathbb {V}(E_i)\!+\!\frac{(1-\lambda )^2}{d^2}\sum _{i\ne j}^d\text {cov}\big (E_i,E_j\big ). \end{aligned}$$

But due to *Law of Total Variance*, $\mathbb {V}(E_i)=\mathbb {V}(F)-\mathbb {E}\big (V_i\big )$. Hence:

$$\begin{aligned} \mathbb {V}(G)&=\left( \lambda ^2+2\lambda (1-\lambda )+\frac{(1-\lambda )^2}{d}\right) \mathbb {V}(F) - \left( \frac{2\lambda (1-\lambda )}{d}+\frac{(1-\lambda )^2}{d^2}\right) \sum _{i=1}^d\mathbb {E}(V_i) \\&\quad +\frac{(1-\lambda )^2}{d^2}\sum _{i\ne j}^d\text {cov}\big (E_i,E_j\big ) \\&=\left( 1-\frac{(d-1)(1-\lambda )^2}{d}\right) \mathbb {V}(F)-\left( 2\lambda (1-\lambda )+\frac{(1-\lambda )^2}{d}\right) \cdot \mathbb {E}\left( \frac{1}{d}\sum _{i=1}^dV_i\right) \\&\quad +\frac{(1-\lambda )^2}{d^2}\sum _{i\ne j}^d\text {cov}\big (E_i,E_j\big ). \end{aligned}$$

But, due to the *Cauchy-Schwarz inequality*, we have that

$$\text {cov}\big (E_i,E_j\big ) \le \sqrt{\mathbb {V}(E_i)\cdot \mathbb {V}(E_j)} \le \mathbb {V}(F),$$

where for the last inequality we have used that $\mathbb {V}(E_i)\le \mathbb {V}(F)$ as implied by the Law of Total Variance. Therefore

$$\begin{aligned} \mathbb {V}(G)&\le \left( 1-\frac{(d-1)(1-\lambda )^2}{d}\right) \mathbb {V}(F)-\alpha \left( 2\lambda (1-\lambda )+\frac{(1-\lambda )^2}{d}\right) \mathbb {V}(F) \\&\quad +\frac{(1-\lambda )^2}{d}(d-1)\mathbb {V}(F)\\&=\left( 1-\alpha (1-\lambda )\frac{1+(2d-1)\lambda }{d}\right) \mathbb {V}(F); \end{aligned}$$

Finally, a simple calculation shows that the factor multiplying $\mathbb {V}(F)$ above is minimized at $\lambda =(d-1)/(2d-1)$, from which the theorem follows. $\square$

### Proof of Theorem 4.2

Let $H:\mathbb {R}_+\rightarrow \mathbb {R}$ be the function defined as

$$\begin{aligned} H:=\frac{\mathcal {L}_{c_1}f_1*\cdots *\mathcal {L}_{c_k}f_k}{f_1*\cdots *f_k}. \end{aligned}$$

For each $1\le i\le k$, let $\varphi _i$ be the Laplace transform of $\tau _i$; in particular, $g_i:=\mathcal {L}_{c_i}f_i/\varphi _i(c_i)$ is a p.d.f. supported on $[0,+\infty )$, and

17 $$\begin{aligned} H=\frac{g_1*\cdots *g_k}{f_1*\cdots *f_k}\prod _{i=1}^k\varphi _i(c_i). \end{aligned}$$

Since both the numerator and denominator correspond to the p.d.f.’s of a sum of *k* non-negative continuous random variables, they are each measurable and almost surely strictly positive and finite. As a result, *H* is a measurable function.

To complete the proof, it suffices to show that

18 $$\begin{aligned} \int _{(t_1,\ldots ,t_k)\ge 0:\,\sum \limits _{i=1}^kt_i\le a} e^{-\sum \limits _{i=1}^kc_i t_i} \prod _{i=1}^kf_i(t_i)\,dt_i =\int _0^a H(t)\,(f_1*\cdots *f_k)(t)\,dt, \end{aligned}$$

for all $a\ge 0$. But this is rather direct because

$$\begin{aligned} \int _0^a H(t)\,(f_1*\cdots *f_k)(t)\,dt\qquad \qquad \qquad \qquad \qquad \qquad \qquad \qquad \qquad \qquad \qquad \qquad \qquad \qquad \end{aligned}$$

$$\begin{aligned}&=\int _0^a (\mathcal {L}_{c_1}f_1*\cdots *\mathcal {L}_{c_k}f_k)(t)\,dt \\&=\int _0^a\,dt\,\int _{(t_1,\ldots ,t_{k-1})\ge 0:\,\sum \limits _{i=1}^{k-1}t_i\le t} e^{-c_k\big (t-\sum \limits _{i=1}^{k-1}t_i\big )}f_k\!\!\left( t-\sum \limits _{i=1}^{k-1}t_i\right) \prod _{i=1}^{k-1} e^{-c_i t_i} f_i(t_i)\,dt_i \\&=\int _0^a\,dt\,\int _{(t_1,\ldots ,t_{k-1})\ge 0:\,\sum \limits _{i=1}^{k-1}t_i\le t} e^{-\sum \limits _{i=1}^{k-1}c_i t_i-c_k\big (t-\sum \limits _{i=1}^{k-1}t_i\big )}f_k\!\!\left( t-\sum \limits _{i=1}^{k-1}t_i\right) \prod _{i=1}^{k-1} f_i(t_i)\,dt_i. \end{aligned}$$

The identity in (18) follows from the Lebesgue-measure-preserving change of variables: $(t_1,\ldots ,t_{k-1},t)\longrightarrow (t_1,\ldots ,t_{k-1},t_d)$, where $t_d:=t-\sum _{i=1}^{k-1}t_i$, thereby completing the proof.
